# Cases in Practice

**Published:** 1877-02-20

**Authors:** Geo. A. Dyer

**Affiliations:** Washington, Ind.


THE
### SOUTHERN MEDICAL RECORD.

VOL. VII. ATLANTA, GA., FEBRUARY 20, 1877. NO. 2.

THOMAS S. POWELL, M. D., EDITOR.
W. T. GOLDSMITH, M. D., Assistant Editor.
R. C. WORD, M. D., Assistant Editor and Business Manager.

SUBSCRIPTION: TWO DOLLARS PER ANNUM, IN ADVANCE.

☞ All communications, and letters on business connected with THE RECORD for 1877 must be addressed to the Business Manager.

### Original and Selected Articles.

### CASES IN PRACTICE.

BY GEO. A. DYER, M. D., WASHINGTON, IND.

1. *A Case of Cerebro Spinal Meningitis, with Treatment.*—June 25, 1875, was called to see Mrs. C., a young married woman, about five months gone. She was suffering from spasms, with violent opisthotonos, headache, pain in left side, rational. Had a chill last night. Pulse 96, skin moderately soft, great heat at base of brain, and in neck. Diagnosed cerebro-spinal meningitis. I gave her immediately a good dose of calomel, with com. extract of colocynth. Ordered free cupping on back of neck and between shoulders. Gave—alternating every three hours—tr. gelseminum, spts. nitre, and tr. hyoscianus, combined with bromide potassium, chloral hydrate, fluid extract of ergot, and morphia. Whisky *ad libitum.*

On the 25th she lost power of speech, and was quite deaf. On the 26th, could read writing, and answer by signs, but was still deaf. Convulsions not so frequent, no opisthotonos, face much flushed, pulse 96, tongue cleaner, the medicine given day before yesterday, followed by oil, having done its work effectually. Gave her, since last night, thirty grains of quinine in six doses, a dose every three hours, to prevent another chill. P. M., 5 o'clock—can talk some; tongue still better; no roaring from quinine. Continued the alternating medicines.

27th. — Light headache, no fever, pulse 96, convulsions once-in-a-while, speech thick.

28th.—Iodide potassium was given in three grain doses every three hours, to promote absorption of fluids. Blisters behind her ears, horseradish leaves to limbs; countenance dull, and expression anxious; whisky freely. In evening speech better, deglutition difficult, but cannot hear yet—pulse 88.

29th.—Pulse 84. No pain except in head. To-day, at 3 P. M., she had very severe spasms, but under application of blister to back of neck, she rallied.

30th.—She seems better in many respects—in fact from this day she began to convalesce, and on the 4th of July was able to sit up.

What is remarkable about this case is the continuance of the spasms ; but I attributed this to her general nervous temperament. Another thing stranger, that with all the spasms, so violent and long continued, she did not miscarry, but, a few months afterwards, I attended her in confinement, and her baby was a fine fat girl, who has not had a day's sickness to this time, December 7, 1876.

A few weeks ago I attended this same lady for tonsillitis. She had marked symptoms of return of the old disease, but I broke up the symptoms almost immediately with a few doses of fluid extract of ergot, which seemed to quiet her brain as if by magic.

2. *A Case of Inguinal Hernia about the Eighth Month of Pregnancy.*—On the evening of the 14th January, 1875, was called to see Mrs. K., a young married woman, pregnant about eight months, who thought she was about to be confined.

She was suffering with pains in her stomach, and from vomiting and straining. Upon examination, I found the os uteri perfectly closed, the plug firm, and no sign of labor. Supposing the symptoms to be those that occur about the eighth month, *settling of the womb*, I gave her an anodyne which gave some relief, and after remaining two hours, as she seemed easy, I went home. The next evening I was again called, the same symptoms appearing, when, upon again examining her, I found quite a large tumor in the right labium. I raised her hips, made taxis, and after a while succeeded in returning an inguinal hernia. I gave her some castor oil, and waited for its action. It soon operated and she became better. I gave her an anodyne, and when she was perfectly easy I went home. When I first saw her, she said she felt the movements of the fœtus, and, therefore, I did not think it necessary to examine as to its condition. Some week or two before this, Mrs. K. had been moving. Contrary to the advice of friends, she had helped to lift stoves, beds, etc., and had even *alone* put down a carpet.

About three or four weeks after I saw her, she was delivered of a still-born babe, and it seemed to have been dead for some time.

It may be that her exertions, at the time of moving and arranging her house, caused the child to turn and get entangled in the cord, as this was found to be its condition.

Mrs. K. was confined again in January, 1876, but the hernia has never since returned.

A simple bandage, tightly applied, seemed to be sufficient in her case, to prevent its return in 1875.

